# Ortho Isomeric Mn(III) N-Alkyl- and Alkoxyalkylpyridylporphyrins—Enhancers of Hyaluronan Degradation Induced by Ascorbate and Cupric Ions †

**DOI:** 10.3390/ijms22168608

**Published:** 2021-08-10

**Authors:** Katarína Valachová, Peter Rapta, Nuno M. M. Moura, Ines Batinic-Haberle, Ladislav Šoltés

**Affiliations:** 1 Centre of Experimental Medicine, Institute of Experimental Pharmacology and Toxicology, Slovak Academy of Sciences, Dúbravská cesta 9, SK-841 04 Bratislava, Slovakia; exfasolt@savba.sk; 2 Institute of Physical Chemistry and Chemical Physics, Slovak University of Technology in Bratislava, Radlinského 9, SK-812 37 Bratislava, Slovakia; 3 LAQV-REQUIMTE, Department of Chemistry, University of Aveiro, 3810-193 Aveiro, Portugal; nmoura@ua.pt; 4 Department of Radiation Oncology, Duke University School of Medicine, Durham, NC 27710, USA; ibatinic@duke.edu

**Keywords:** SOD mimics, Mn porphyrins, hyaluronic acid degradation, ascorbate/copper, relevance to cancer, ROS production

## Abstract

High levels of hyaluronic acid (HA) in tumors correlate with poor outcomes with several types of cancers due to HA-driven support of adhesion, migration and proliferation of cells. In this study we explored how to enhance the degradation of HA into low-molecular fragments, which cannot prevent the immune system to fight tumor proliferation and metastases. The physiological solution of HA was exposed to oxidative degradation by ascorbate and cupric ions in the presence of either one of three *ortho* isomeric Mn(III) substituted *N*-alkyl- and alkoxyalkylpyridylporphyrins or *para* isomeric Mn(III) *N*-methylpyridyl analog, commonly known as mimics of superoxide dismutase. The changes in hyaluronan degradation kinetics by four Mn(III) porphyrins were monitored by measuring the alteration in the dynamic viscosity of the HA solution. The *ortho* compounds MnTE-2-PyP^5+^ (BMX-010, AEOL10113), MnTnBuOE-2-PyP^5+^ (BMX-001) and MnTnHex-2-PyP^5+^ are able to redox cycle with ascorbate whereby producing H_2_O_2_ which is subsequently coupled with Cu(I) to produce the ^•^OH radical essential for HA degradation. Conversely, with the *para* analog, MnTM-4-PyP^5+^, no catalysis of HA degradation was demonstrated, due to its inertness towards redox cycling with ascorbate. The impact of different Mn(III)-porphyrins on the HA decay was further clarified by electron paramagnetic resonance spectrometry. The ability to catalyze the degradation of HA in a biological milieu, in the presence of cupric ions and ascorbate under the conditions of high tumor oxidative stress provides further insight into the anticancer potential of redox-active *ortho* isomeric Mn(III) porphyrins.

## 1. Introduction

**Figure 1:**
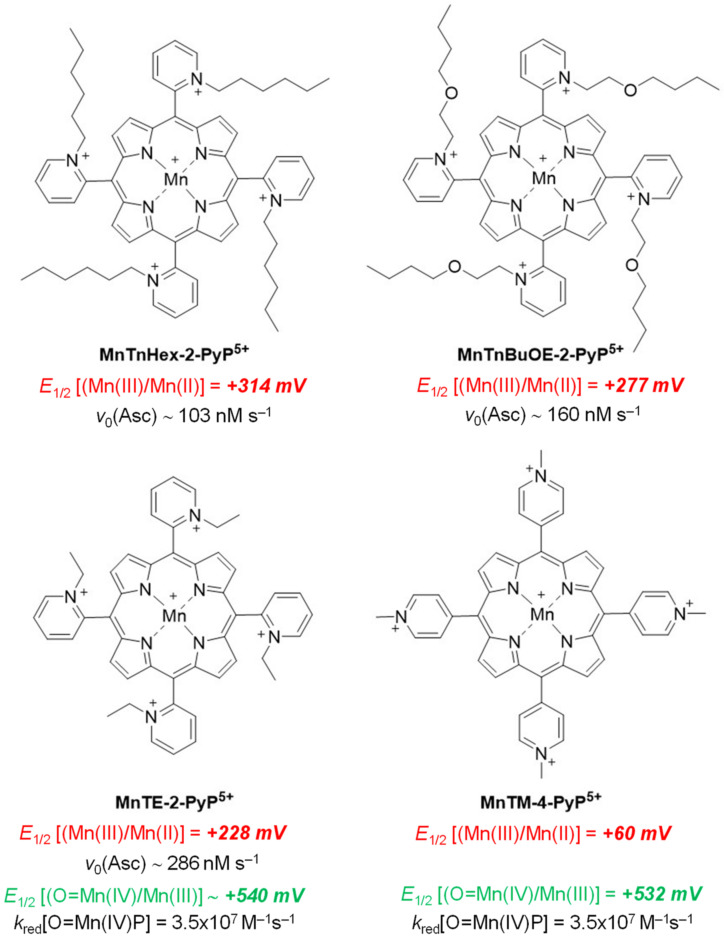
Chemical structures of redox-active cationic Mn(III) porphyrins and the reduction potentials, *E*_1/2_ vs. NHE for Mn^III^/Mn^II^ (in red) and O = Mn^IV^/Mn^III^ (in green, measured at pH 11) redox couples [6,7]. The values shown for the *k*_red_ and *E*_1/2_ [(O = Mn(IV)/Mn(III)] for the *ortho* analog, MnTE-2-PyP^5+^, are based on the reported values [6] for the methyl analog, MnTM-2-PyP^5+^. We may safely assume that those values for methyl and ethyl analogs are similar based on the fact that kinetic and thermodynamic values reported thus far for the methyl and ethyl analogs are similar, if not identical [7]. Values for pH 11 are shown to indicate that *E*_1/2_ values are identical for all isomers. The values for O = Mn(IV)/Mn(III) couple were obtained in the whole range of pH only for MnTE-2-PyP [8] and at pH = 7 it is +517 mV vs. NHE [2]. Additionally, the initial ascorbate oxidation rates, *v*_0_(Asc), which relate to the ability of Mn porphyrins to oxidize ascorbate to ascorbyl radical employing Mn^III^/Mn^II^ redox couple were reported in [1], and the second order rate constants *k*_red_[O = Mn(IV)P] at 37 °C for the reduction of the high valent oxo Mn(IV) state by ascorbate were reported in [6].

Mn(III) cationic *ortho* substituted *N*-alkyl- and alkoxyalkylpyridylporphyrins (MnPs) were initially designed as superoxide dismutase mimics, i.e., the catalysts of O_2_^•−^ dismutation. As the knowledge of their chemistry increased along with the rising knowledge of redox biology of a cell, it became obvious that such compounds undergo diverse reactions in biological systems and carry large therapeutic potential [1,2]. Two compounds, Mn(III) *meso*-tetrakis(*N*-ethylpyridinium-2-yl)porphyrin, MnTE-2-PyP^5+^ (AEOL10113, BMX-010) and Mn(III) *meso*-tetrakis(*N*-butoxyethylpyridinium-2-yl) porphyrin, MnTnBuOE-2-PyP^5+^ (BMX-001, Figure 1) are now in five phase two clinical trials [1,3]. Most of the potent superoxide dismutase (SOD) mimics have Mn in a +3 oxidation state. Such a state is stabilized by the porphyrin macrocyclic ring, which also assures the integrity of the Mn center, preventing its loss from the porphyrin ligand [1]. Based on the favorable redox properties, we aim here to see if MnTE-2-PyP^5+^ and MnTnBuOE-2-PyP^5+^, as well as Mn(III) *meso*-tetrakis(*N*-n-hexylpyridinium-2-yl)porphyrin, MnTnHex-2-PyP^5+^, can assist Cu/ascorbate system in degrading high-molar-mass hyaluronic acid (HA). We compared these *ortho* (2) isomers to a *para* (4) isomer, Mn(III) *meso*-tetrakis(*N*-methylpyridinium-4-yl)porphyrin, MnTM-4-PyP^5+^ (Figure 1). Relative to *ortho* isomers, MnTM-4-PyP^5+^ compound is stabilized in a +3 oxidation state, and cannot be easily reduced with cellular reductants due to its more than 160 mV negative metal-centered reduction potential for Mn(III)/Mn(II) redox couple, *E*_1/2_ = +60 mV vs. NHE when compared to *ortho* analogs [3,4]. Namely, the MnTE-2-PyP^5+^ and MnTnBuOE-2-PyP^5+^ have *E*_1/2_ of +228 mV vs. NHE and +277 mV vs. NHE for Mn(III)/Mn(II) redox couples, respectively, [1]. Due to inferior redox properties, MnTM-4-PyP^5+^ cannot be readily reduced with ascorbate in order to become oxidized with oxygen in the second step to produce O_2_^•−^ and, subsequently, (enzymatically or via self-dismutation) H_2_O_2_. While the mechanism of the catalysis of O_2_^•−^ dismutation by *ortho* isomers and *para* MnTM-4-PyP^5+^ is identical, the thermodynamic yields controlled by their reduction potentials are different. Importantly, *ortho* compounds are bulky and more so those with long hexyl and butoxyethyl lipophilic chains. *Para* isomer with short methyl chains, MnTM-4-PyP^5+^, is planar and that facilitates its interactions with biomolecules such as intercalation into nucleic acids, which results in the loss of its SOD-like activity [5].

Ascorbic acid (AscH_2_)—vitamin C—is an essential nutritional component for humans [9]. At physiological pH, ascorbic acid is in monodeprotonated form, AscH^−^. In vivo, besides other physiological functions, ascorbate acts as an antioxidant; one of its major roles, in concert with tocopherol, is the protection of the lipid membranes against peroxidation. Yet, in the presence of transition metals such as Cu and Fe, ascorbate becomes involved in oxidative processes. Under aerobic physiological conditions, ascorbate can reduce Cu(II) to Cu(I) while being oxidized to ascorbyl radical, HAsc^•^ [10]. Cu(I) subsequently reacts with H_2_O_2,_ producing hydroxyl radicals while it becomes reoxidized/regenerated into Cu(II), closing a catalytic cycle; the catalysis continues as long as ascorbate and oxygen are available.

The system is known as Weissberger’s biogenic oxidative system (WBOS) [11] and is reportedly one of the most effective generators of ^•^OH radicals, which are responsible for the destruction of DNA, RNA, proteins, lipids, and polysaccharides in living organisms [12]. Such an ^•^OH-generating system may be employed in exploring the antioxidative efficacy of natural or synthetic compounds for therapeutic and industrial purposes [11,13].

On the outer surface of the cell, mammalian cells have a highly specific glycocalyx, which is non-immunogenic for components of the immune system. We can assume that glycocalyx of the cancer cells, including the metastatic ones, is somewhat different from that of normal cells [14,15]. Consequently, a different glycocalyx in metastatic and cancer cells could become immunogenic [16]. However, the reported data show that the surface of the cancer cells is populated with CD44 receptors, which tightly bind high-molar-mass hyaluronic acid extruding from cells [17]. Consequently, the cancer cell, enveloped with non-immunogenic high-molar-mass HA, cannot be recognized by the immune system.

Therefore, our attention has been focused on developing strategies to destruct the “invisibile” coat of the cancer cells by radical-based HA degradation, using WBOS further enforced by MnPs. This should lead to the effective shortening of the long chains of HA macromolecule towards the formation of the fragments of low-molecular HA mass. Such fragments would become immunogenic, attracting components of the immune system to reach the surface of the cancer cell, recognize a foreign glycocalyx and in turn, destruct both cancer and metastatic cells.

In this manuscript we investigate the oxidative degradation of HA catalyzed by Mn porphyrins in the presence of Cu(II) and ascorbate, using three *ortho* isomeric cationic Mn porphyrins (MnTE-2-PyP^5+^, MnTnHex-2-PyP^5+^, and MnTnBuOE-2-PyP^5+^) and compare them to the efficacy of the less redox active *para* isomer, MnTM-4-PyP^5+^. A general scheme of their action in the presence of WBOS is proposed. Different behavior of investigated Mn complexes—which can act as catalysts of HA oxidative degradation as well as scavengers of alkoxy-/peroxy type HA macroradicals—is discussed.

## 2. Results and Discussion

**Figure 2:**
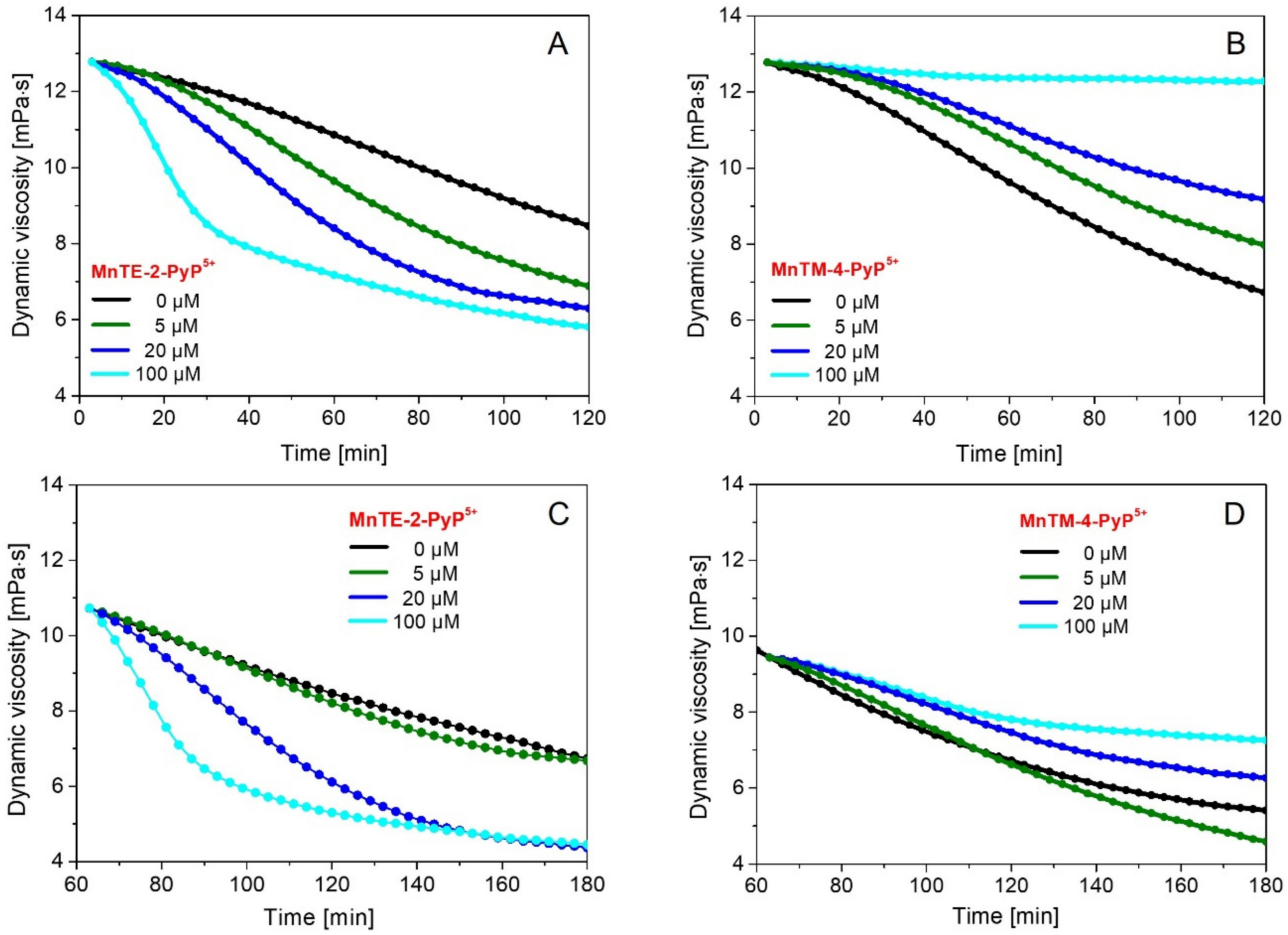
Time-dependent changes in dynamic viscosity of the HA solution subjected to 1 µM Cu(II) and 100 µM ascorbate in the presence of (**A**) MnTE-2-PyP^5+^ or (**B**) MnTM-4-PyP^5+^ at different concentrations: 0 (black curve), 5 (green curve), 20 (blue curve), and 100 µM (cyan curve). The concentration of HA was 1.75 mg/mL. Panel (**C**)—MnTE-2-PyP^5+^ was added at 1 h after HA degradation began. Panel (**D**)—MnTM-4-PyP^5+^ was added at 1 h after HA degradation began.

When the HA was subjected to oxidative degradation by 1 µM Cu(II) and 100 µM ascorbate, significant degradation of HA macromolecules by ^•^OH radicals was observed (Figure 2A, black curve, the reference). The addition of MnTE-2-PyP^5+^ at concentrations of 0, 5, 20 and 100 µM resulted in dose-dependent enhancement of HA degradation. The highest rate of HA degradation occurred within 30 min and at 100 µM MnP and was subsequently slowed down due to the consumption of oxygen and ascorbate (Figure 2A, cyan curve).

When MnTE-2-PyP^5+^ was added to the HA reaction mixture at 20 and 100 µM (but not at low 5 µM concentration) at 1 h after the production of alkoxy-/peroxy-type radicals was already established [18], again a large rate of HA degradation was initially observed and was subsequently reduced due to the consumption of oxygen and ascorbate (see Figure 2C, blue and cyan curve, respectively). The increased degradation of HA may be due to the reduction of MnP with ascorbate, but also to the reaction of Mn porphyrins with peroxy- and alkoxy-type radicals, producing high-valent and highly oxidizing Mn(IV) and Mn(V) oxo species. These high-valent oxo species may undergo reduction with ascorbate and could enhance the HA degradation. 

However, with the *para* isomer, MnTM-4-PyP^5+^, we observed a different behavior (Figure 2B). When used at 100 µM (where MnTE-2-PyP^5+^ is the most potent enhancer of HA degradation), no HA degradation was demonstrated (see Figure 2B, cyan curve). HA degradation was inhibited to a lower degree in the presence of 20 µM MnTM-4-PyP^5+^ (blue curve), and more so in 5 µM MnTM-4-PyP^5+^ (green curve). We explain such data with the differences in the MnP/ascorbate ratio. When the ratio was 1, no reduction of Mn^III^P^5+^ with ascorbate happened; in turn, no reoxidation of Mn^II^P^4+^ occurred that would have given rise to O_2_^•−^ and subsequently H_2_O_2_. However, when ascorbate is in 5 or 20-fold excess over MnP, the reduction of MnTM-4-PyP^5+^ with ascorbate becomes thermodynamically permissible, apparently giving rise to lower inhibition of HA degradation. The corresponding initial rates, −(d*η*/d*t*)*_t_*_=0_, are listed in Table 1.

In conclusion, due to the lack of Mn(III)/Mn(II) redox cycling with ascorbate and thus to the lack of H_2_O_2_ production essential for HA degradation, the *para* MnTM-4-PyP^5+^ is an inefficient catalyst. Regardless of the time when MnP was added into the reaction mixture, the data point to the critical role the reduction of Mn^III^P^5+^ with ascorbate plays, as its subsequent reoxidation is accompanied with the production of H_2_O_2_.

**Figure 3:**
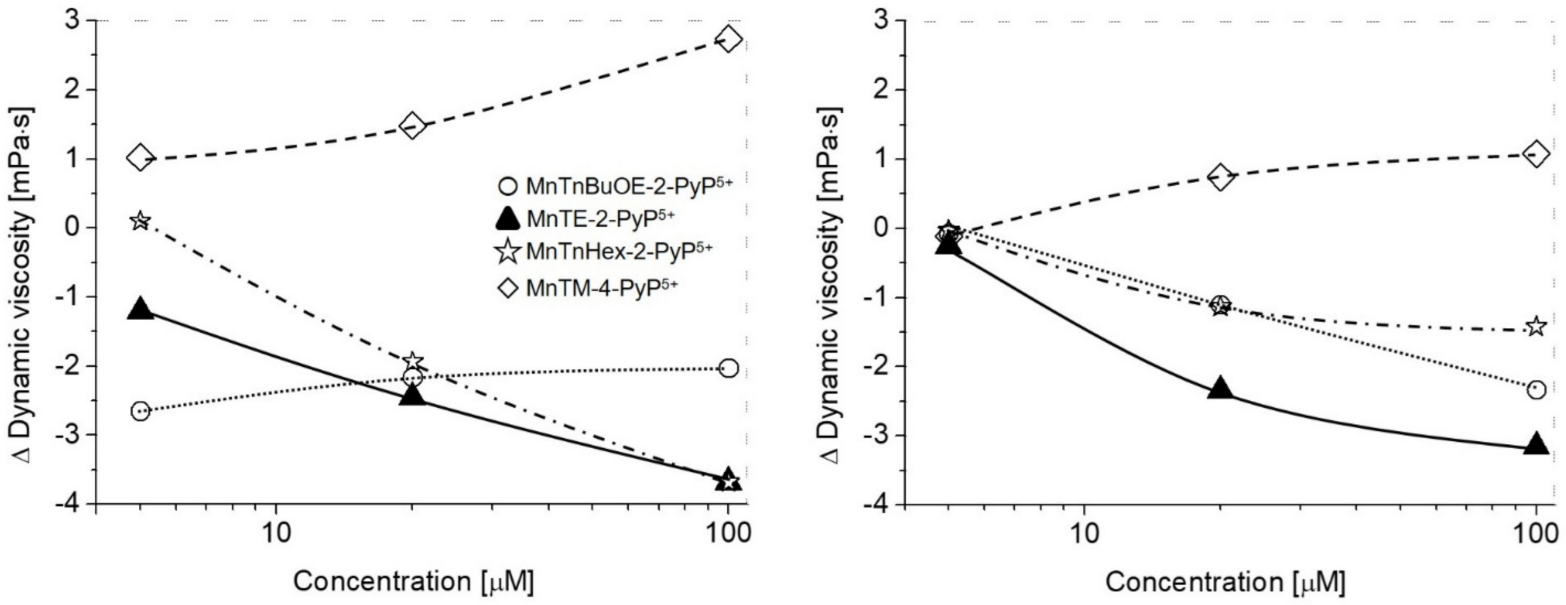
Concentration-dependent changes in dynamic viscosity of HA solutions exposed to oxidative degradation by Cu(II) (1 µM) and ascorbic acid (100 µM) in the presence of MnTnBuOE-2-PyP^5+^ (circles, dotted lines), MnTE-2-PyP^5+^ (triangles, solid lines), MnTnHex-2-PyP^5+^ (stars, dash-dotted lines) or MnTM-4-PyP^5+^ (diamond, dashed lines) at concentrations of 5, 20 and 100 µM. The concentration of HA was 1.75 mg/mL. **Left panel**—MnPs were added to the HA mixture before initiating HA degradation. The 60 min data are shown. **Right panel**—MnPs were added to the HA mixture 1 h later. The 120 min data are shown.

Additionally, we compared activity of four investigated Mn porphyrins at two time points in Figure 3: 60 min (left panel) and 120 min (right panel). The dose-dependent HA degradation at 60 min was seen with MnTE-2-PyP^5+^ (triangle) and MnTnHex-2-PyP^5+^ (star), but not with MnTnBuOE-2-PyP^5+^ (circle). The fastest degradation of HA occurred at the concentration of 5 µM, where the large excess of ascorbate over MnP favored the reduction of MnP. The three *ortho* isomeric Mn(III)Ps are easily reducible with ascorbate. When cycling back to Mn(III)P with oxygen, these MnPs would give rise to H_2_O_2_ which in turn would react with Cu(I) to make the hydroxyl radical. MnTM-4-PyP^5+^ (diamond), however, cannot be easily reduced with ascorbate (*E*_1/2_(Asc^•−^, H^+^/AscH^−^) = +282 mV vs. NHE) unless ascorbate is in excess. Thus MnTM-4-PyP^5+^ and ascorbate cannot contribute to the hydroxyl radical production via reoxidation with oxygen to the same extent as *ortho* analogs do. MnTE-2-PyP^5+^ (triangle) is the most potent promoter of alkoxy-/peroxy-type-induced HA degradation at 120 min (Figure 3, right panel). Under these conditions, dose-dependence was seen also with both MnTnBuOE-2-PyP^5+^ (circle) and MnTnHex-2-PyP^5+^ (star). In the presence of MnTnHex-2-PyP^5+^ (star), the rate of HA degradation was lower than the rate obtained in the presence of the two other complexes.

The long alkyl chains impose steric hindrance towards the reaction with ascorbate. While MnTnBuOE-2-PyP^5+^ has equally long pyridyl substituents as MnTnHex-2-PyP^5+^, the oxygen atoms facilitate the approach of ascorbate to the Mn center, presumably via hydrogen bonding between the oxygen atoms of butoxyethyl groups and the hydrogen atoms of ascorbate. In turn, it is a better catalyst of ascorbate oxidation. The ascorbate oxidation rate, *v*_0_ = 103 nM s^−1^, is the lowest for MnTnHex-2-PyP^5+^, followed by *v*_0_ = 160 nM s^−1^ for MnTnBuOE-2-PyP^5+^ and *v*_0_ = 286 nM s^−1^ for MnTE-2-PyP^5+^ [1,19].

**Figure 4:**
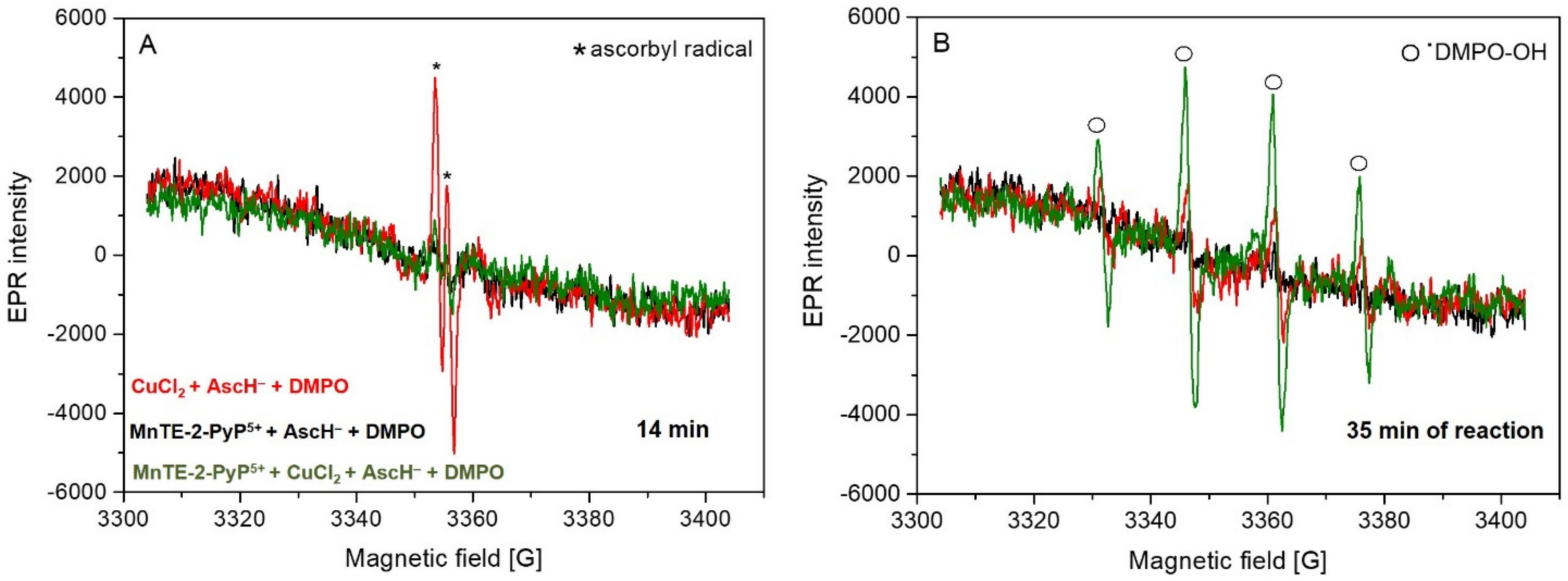
EPR spectra monitored after (**A**) 14 min and (**B**) 35 min of reaction in the aqueous solutions saturated with oxygen at room temperature for the following systems: *System 1* (black traces): 7.5 μM MnTE-2-PyP^5+^ + 0.75 mM AscH^−^ + 0.053 M DMPO; *System 2* (red traces): 7.5 μM CuCl_2_ + 0.75 mM AscH^−^ + 0.053 M DMPO; *System 3* (green traces): 7.5 μM MnTE-2-PyP^5+^ + 7.5 μM CuCl_2_ + 0.75 mM AscH^−^ + 0.053 M DMPO); (*—ascorbyl radical, ○—^•^DMPO-OH adduct).

**Figure 5:**
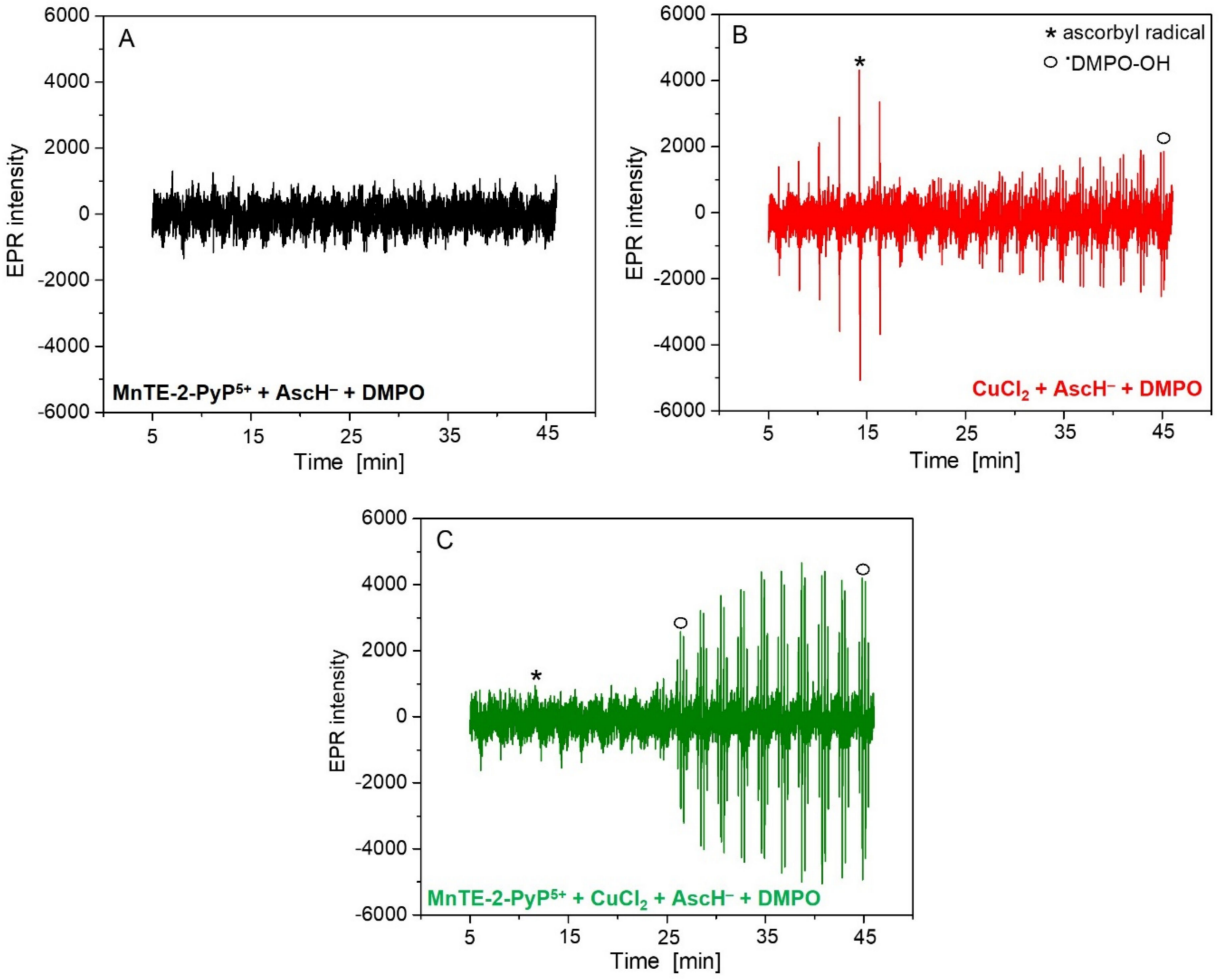
EPR spectra (20 spectra were measured, each consists of 3 scans) measured for (**A**) *System 1*, (**B**) *System 2* and (**C**) *System 3* as described in Figure 5. (*—ascorbyl radical, ○—^•^DMPO-OH adduct).

EPR spectroscopy using a spin trapping technique was applied to prove the proposed reaction mechanism, as shown in Figure 4 and Figure 5. For the reaction system (in the absence of HA) containing MnTE-2-PyP^5+^ and ascorbate, a low level of reactive radicals was detected in aqueous solutions in the presence of 5,5-dimethyl-1-pyrroline *N*-oxide (DMPO, black lines in Figure 4 and Figure 5A). When using CuCl_2_ instead of MnTE-2-PyP^5+^, at first a strong increase in ascorbyl radical was observed, which was followed by a continuous slight increase in ^•^DMPO-OH adducts (red lines in Figure 4 and Figure 5B).

**Figure 6:**
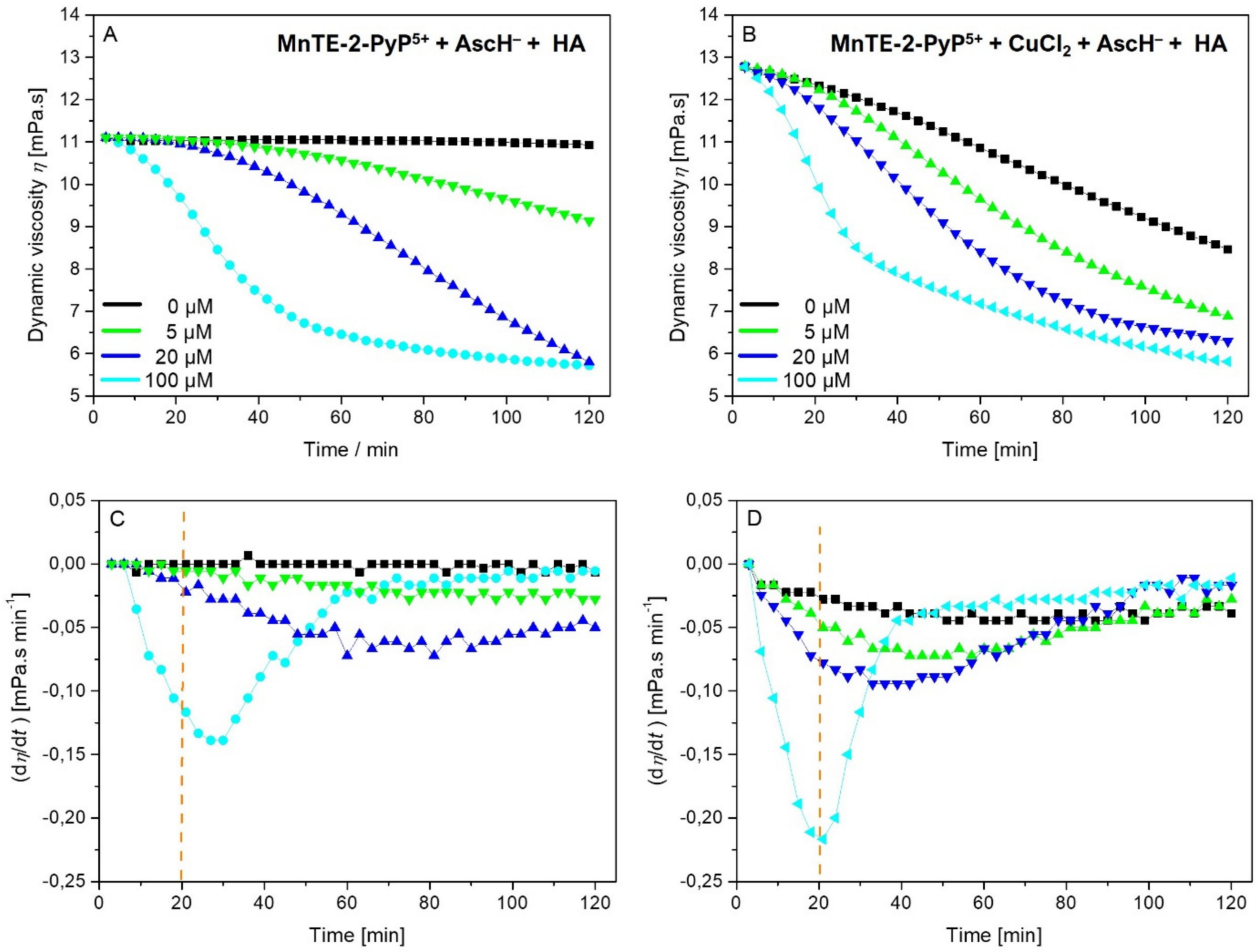
Time-dependent changes in dynamic viscosity (*η*) of the HA solution subjected to MnTE-2-PyP^5+^ at concentrations 0 (black curve), 5 (green curve), 20 (blue curve), and 100 µM (cyan curve) and 100 µM ascorbate in the absence of Cu(II) (**A**) and in the presence of 1 µM Cu(II) (**B**), as well as the corresponding first derivative curves in time (d*η*/d*t* = f(*t*)) in the absence of Cu(II) (**C**) and in the presence of 1 µM Cu(II) (**D**). The HA concentration was 1.75 mg/mL.

This unambiguously confirms the enhancement in the production of reactive oxygen species (ROS) in the reaction system containing MnTE-2-PyP^5+^, ascorbate and Cu(II). Thus, Figure 6A demonstrates that a MnP/Asc system catalyzes HA degradation. The catalysis is further enhanced in the presence of Cu(II) within 10 to 30 min of the main degradation phase (Figure 6B). The initial rates at 20 min of reaction were calculated from the first derivation of the time-dependent changes in dynamic viscosity (*η*) of the HA solution. The solution comprised MnTE-2-PyP^5+^, and 100 µM ascorbate in the absence (see Figure 6C) and presence of 1 µM Cu(II) (see Figure 6D). The corresponding rates at *t* = 20 min, (defined as –(d*η*/d*t*)*_t_*_=20_), are listed in Table 2. The enhanced production of ROS was further demonstrated by the EPR spin trapping technique for system MnTE-2-PyP^5+^ + AscH^−^ + Cu(II) at much lower concentrations of reagents in the presence of HA [20].

**Scheme 1:**
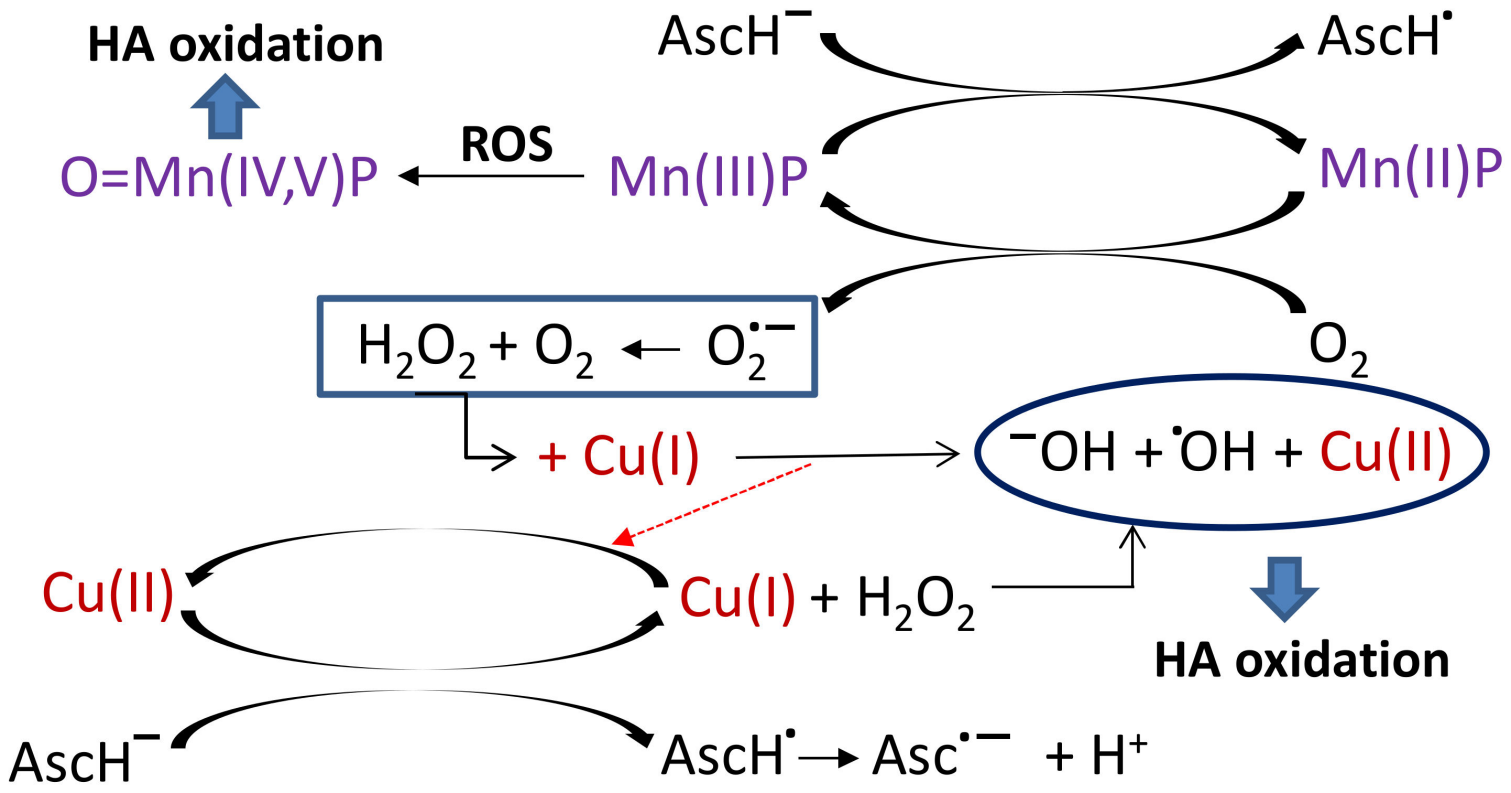
Proposed mechanism of hyaluronic acid degradation in the presence of *ortho* isomeric Mn(III)-porphyrins, Cu(II) and ascorbate giving rise to enhanced production of ^•^OH radicals and HA oxidation (note that the formation of hydroperoxyl radical HO_2_^•^ is valid for acidic environment while at physiological pH 7.8 the superoxide anion dominates. During inflammation, which accompanies cancer, the tissues are in a slightly acidic environment [26]. The bottom part of Scheme 1 represents WBOS.

The following mechanism, depicted in Scheme 1, for the catalysis of HA degradation was proposed. The redox cycling with ascorbate and oxygen allows *ortho* isomers to produce O_2_^•−^ in the reoxidation step, which would dismutate (through self-dismutation or enzymatically) to H_2_O_2_. The subsequent reaction of H_2_O_2_ with ascorbate-reduced Cu(I) would give rise to a ^•^OH radical and would cause oxidative degradation of HA (Scheme 1). In such a scenario, *ortho* Mn porphyrins, by producing the additional amount of H_2_O_2_ and in turn ^•^OH radicals, act as catalysts of HA oxidative degradation. HA is present at high levels in tumors and is reported to promote carcinogenesis and metastases [17,21]. Our studies taught us that *ortho* Mn porphyrins are able to promote the HA degradation in the presence of Cu(II) [or Fe(III)] and ascorbate. Such knowledge increases our insight into their anticancer therapeutic potential [22,23,24,25].

The participation of transition metals Cu(II) [or Fe(III)] in free radical generation in the presence of ascorbate under aerobic conditions is shown by the Haber-Weiss reaction (Equation (1)). In that reaction O_2_^•−^ and H_2_O_2_ interact in the presence of transition metal catalysts *via* the reaction:

O_2_^•−^ + H_2_O_2_ → ^•^OH + OH^−^ + O_2_ (transition metal, Cu or Fe catalyzed) (1)

**Scheme 2:**
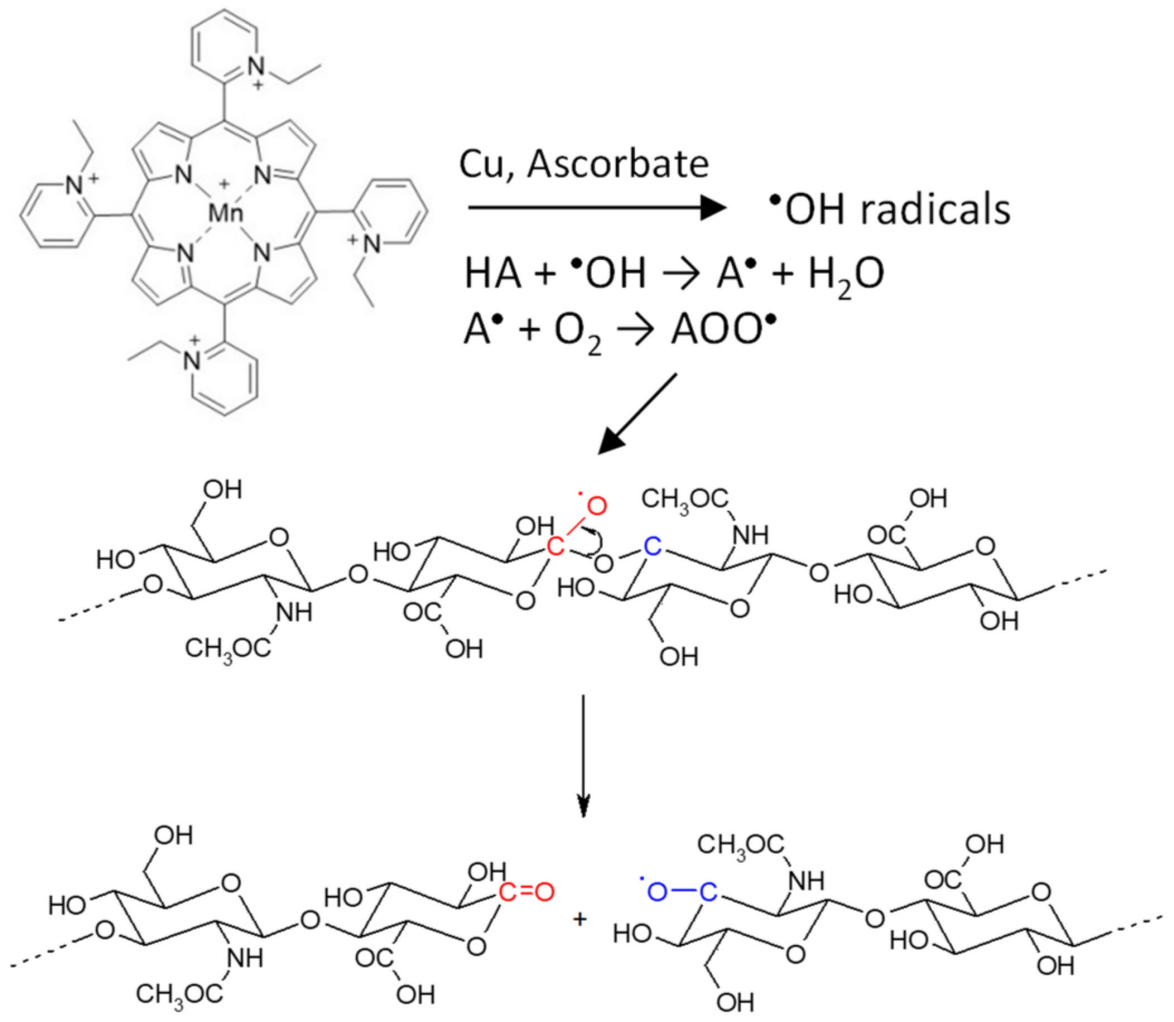
HA degradation by ^•^OH radicals, produced in a reaction mixture containing Cu(II), ascorbate and Mn porphyrins, leading to the polymer fragments of lower molecular weight as e.g., an alkoxy-type macroradical and a HA-like macromolecule bearing a terminal C=O group.

The HA degradation by ^•^OH radicals, produced in a reaction mixture containing Cu(II), ascorbate and Mn porphyrins results in polymer fragments of lower molecular weight [11,13,20] as illustrated in Scheme 2. This is a consequence of a reaction of hydroxyl radicals with HA forming carbon centered radicals that react with molecular oxygen, resulting in the generation of peroxyl radicals serving as a source of ^•^OOA peroxyl radicals and consequently a source of ^•^DMPO-OA adducts in spin trapping studies [20,27].

**Figure 7:**
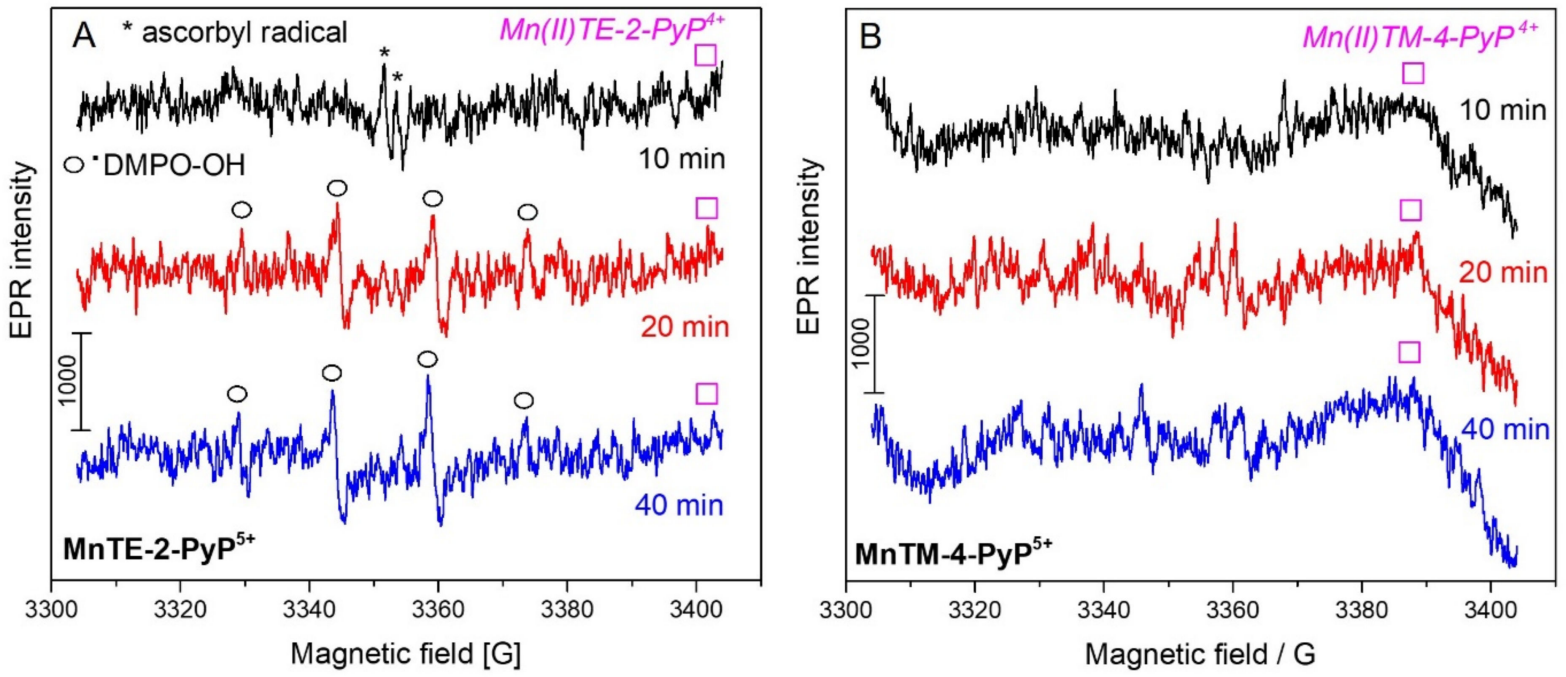
Time evolution of EPR spectra of DMPO spin adducts and of *ortho* Mn(II)TE-2-PyP^4+^ (**A**) and *para* Mn(II)TM-4-PyP^4+^ (**B**) state in the aqueous solutions saturated with air at room temperature for the system: 250 μL water solution containing 1 μM Cu(II), 100 μM Mn(III)P, 1.75 mg/mL HA and 100 μM AscH^−^ + 50 μL DMPO (25 μL DMPO/1 mL H_2_O) for MnTE-2-PyP^5+^ (**A**) and for MnTM-4-PyP^5+^ (**B**) (*—ascorbyl radical; **○**—^•^DMPO-OH adduct; χ—signal from Mn(II) state).

While the same mechanism as shown in Scheme 1 with regards to ascorbate would operate with both *ortho* and *para* isomers, the reduction of *para* MnTM-4-PyP^5+^ into MnTM-2-PyP^4+^,with ascorbate is not thermodynamically favored due to its more negative *E*_1/2_ for the Mn(III)/Mn(II) redox couple (more than 160 mV) than that of an *ortho* analog. It cannot be easily reduced in order to get reoxidized unless ascorbate is in excess (Figure 1). Moreover, the reduced and planar MnTM-4-PyP^4+^ would intercalate into HA which would prevent its reoxidation with oxygen and production of superoxide/H_2_O_2_ and eventually ^•^OH radical. Thus, MnTM-4-PyP^5+^ would have not given rise to a significant amount of H_2_O_2_ to allow for Cu(I)/H_2_O_2_-driven production of ^•^OH radical. Indeed, neither enhancement of HA degradation nor generation of ^•^OH radical was demonstrated (Figure 7B). 

With MnTE-2-PyP^5+^, a clear production of ROS was observed in the presence of HA within the first 40 min (EPR spectra of ^•^DMPO-OH adducts are marked by circles, Figure 7A). Due to its rapid reoxidation, a significant EPR signal of reduced Mn(II) was not seen (Mn(II) line in Figure 7A). With MnTM-4-PyP^5+^, no ROS formation was observed as a negligible amount of DMPO spin adducts was detected (Figure 7B). Data are in agreement with the differences in kinetics and thermodynamics of the reaction of *ortho* and *para* Mn porphyrins with ascorbate and oxygen [4,19,28]. MnTE-2-PyP^5+^ gets readily reduced with ascorbate (see Figure 1) and the reduced Mn(II) cycles back to Mn(III) state (with *k*_ox_(MnP) ~8 × 10^4^ M^−1^s^−1^ [28]), while reducing oxygen to the superoxide anion radical (see Scheme 1).

Moreover, MnTM-4-PyP^5+^ inhibited HA degradation regardless of the time point at which it was added into the Cu(II)/ascorbate/HA reaction mixture (see Figure 2B). A small enhancement in HA degradation was seen only when the ascorbate (100 µM) was present at a huge excess over MnP (5 µM). Such scenario allowed only for a low extent of MnP reduction and consequently for a very low yield of reoxidation reaction (with *k*_ox_(MnP) ~1.1 × 10^6^ M^−1^ s^−1^).

The behavior of MnTM-4-PyP^5+^ may be explained as follows: as compared to *ortho* isomer*, para* MnTM-4-PyP^5+^ is a planar molecule. It was reported that, when reduced to MnTM-4-PyP^4+^, it loses axially bound molecules and becomes even more planar and thus intercalates readily into nucleic acids (RNA and DNA) [5]. Axially bound water molecules would have otherwise imposed steric hindrance to intercalation. Such interactions resulted in a loss of its SOD-like activity [5]. Once nucleic acids were removed, the SOD-like activity was restored [5]. It may be thus safely assumed that the planar reduced MnTM-4-PyP^4+^ would intercalate into the HA and stabilize the polymer. Being trapped within the HA polymer, the reduced MnTM-4-PyP^4+^ would not be able to cycle back to Mn(III)P with oxygen in order to give rise to reactive species and degrade HA, despite its faster reoxidation rate with oxygen [*k_ox_*(MnP)] than that of MnTE-2PyP^4+^. It is well known that charge/charge interactions, hydrogen bonding and intercalations have a dramatic impact on the in vivo actions of molecules [5,29,30].

## 3. Materials and Methods

### 3.1. Chemicals

Hyaluronan (M_w_ = 1.69 MDa, M_w_/M_n_ = 1.64) was obtained from Lifecore Biomedical Inc., Chaska, MN, USA (content of transition metals: copper <1 ppm, iron 6 ppm). NaCl p.a. and CuCl_2_·2H_2_O p.a. were purchased from Slavus Ltd., Bratislava, Slovakia. Ascorbic acid used was obtained from Merck KGaA, Darmstadt, Germany. Aqueous solutions of MnTE-2-PyP^5+^, 19.6 mM, MnTnHex-2-PyP^5+^, 7.23 mM, and MnTnBuOE-2-PyP^5+^, 5.69 mM were obtained from Batinic-Haberle’s lab, Duke University School of Medicine, North Carolina, USA. MnTM-4-PyP^5+^ was obtained from the University of Aveiro, Aveiro, Portugal and was prepared via alkylation of metal-free ligand followed by its metalation. 5,5-Dimethyl-1-pyrroline *N*-oxide (DMPO, ≥97%) was purchased from Sigma-Aldrich Chemie GmbH, Steinheim, Germany. Deionised high-purity grade water, with conductivity of ≤0.055 µS/cm, was made by using the TKA water purification system (Water Purification Systems GmbH, Niederelbert, Germany).

### 3.2. Preparation of Stock and Working Solutions

The HA samples (16 mg) were dissolved in 0.15 M aqueous NaCl solution for 24 h in the dark in two steps: first, 4.0 mL of 0.15 M NaCl was added to HA swelling and after 6 h, 0.15 M NaCl in the volumes 3.90, 3.85, 3.79 or 3.76 mL was added. Ascorbic acid (16 mM) and cupric chloride solutions (160 µM) were prepared in 0.15 M aqueous NaCl. Solutions of MnTM-4-PyP^5+^ and MnTE-2-PyP^5+^ (16 mM) were made and diluted to concentrations 3.2 and 0.8 mM in deionized water. Solutions of MnTnBuOE-2-PyP^5+^ and MnTnHex-2-PyP^5+^ at concentrations 3.2 and 0.8 mM were made in deionized water. The DMPO solution was prepared by dissolving 25 µL of DMPO (distilled prior to the application and stored at −18 °C) in 1 mL of deionized water.

### 3.3. Studies of Inhibition of Hyaluronan Degradation 

The assays used to explore the effect of the MnPs on oxidatively degraded hyaluronan were as follows:

i. A volume of 50 µL of CuCl_2_ solution was added to the HA solution (7.90, 7.85, 7.79 or 7.76 mL), and the mixture, after a stirring of 30 s, was left to stand for 7.5 min at room temperature. Then, the MnP solution (0, 50, 110 or 140 µL) was added to the reaction mixture, followed by stirring again for 30 s. Finally, 50 µL of ascorbic acid solution was added to the solution, and the mixture was stirred for another 30 s. The solution was then immediately transferred into the viscometer Teflon^®^ cup reservoir.
ii. In the second experimental design, a procedure similar to that described in (**i**) was applied; however, after 7.5 min, the 50 µL of ascorbic acid solution was added and stirred for 1 h. Then 50, 110 or 140 µL of the MnP solution was added, followed by stirring for 30 s. The solution mixture was then immediately transferred into the viscometer Teflon^®^ cup reservoir.
  Dynamic viscosity of the reaction mixture (8 mL) containing HA (1.75 mg/mL), ascorbate (100 μM), Cu(II) ions (1 μM) and Mn porphyrins (final concentrations of 0, 5, 20, 100 μM) was measured by a Brookfield LVDV−II+PRO digital rotational viscometer (Brookfield Engineering Labs., Middleboro, MA, USA) at 25.0 ± 0.1 °C, 180 rpm at a shear rate of 237.6 s^−1^ for 2 h. All details of how the degradation of HA can be assessed by dynamic viscosity is described in [31].

### 3.4. Electron Paramagnetic Resonance (EPR)

The generation of free radicals during HA degradation was examined by a spin trapping technique in an EPR X-band EMX spectrometer (Bruker, Rheinstetten, Germany) at ambient temperature. The reaction mixture was composed of HA solution (1.75 mg/mL), Cu(II) ions (1.0 µM), MnPs (100 µM), and ascorbic acid (100 µM). The spectra were recorded at 2, 20, 60, 90, or 150 min after the addition of ascorbic acid. Each solution (250 µL) was thoroughly stirred with 50 µL of 0.212 M DMPO spin trap prior to its insertion in a thin flat EPR quartz cell. The operational parameters of the equipment were adjusted as follows: center field 3354 G, sweep width 100 G, time constant 81.92 ms, conversion time 20.48 ms, receiver gain 5 × 10^5^, microwave power 10 mW, and modulation amplitude 2 G, number of scans from 3 to 20.

## 4. Conclusions

We have demonstrated herein the enhancement of the Cu(II)/ascorbate-driven oxidative degradation of hyaluronic acid by redox active cationic *ortho* isomeric Mn(III) alkyl-alkoxyalkylpyridylporphyrins, but not by *para* isomer MnTM-4-PyP^5+^. Their catalytic abilities or lack thereof are due to the differences in their thermodynamic and kinetic properties. The reduction of *para* MnTM-4-PyP^5+^ with ascorbate is not thermodynamically favored due to its more negative *E*_1/2_ for the Mn(III)/Mn(II) redox couple than that of an *ortho* analogs and happens only at sufficient excesses of ascorbate. In turn, no reoxidation of reduced Mn(II)TM-4-PyP^4+^ happens which would have otherwise enabled formation of ^•^OH radical. Importantly, the planar *para* MnTM-4-PyP^5+^ and more so when reduced, intercalates into the HA and stabilizes the polymer. In turn, the strongly intercalated reduced complex within the HA network is not easily accessible to molecular oxygen and remains inactive. 

Our data point to another possible *in vivo* action of Mn porphyrins, commonly known as SOD mimics. Due to the high level of HA in cancer (which seems to promote cancer proliferation and metastases), its oxidative degradation catalyzed by MnPs (in the presence of endogenously available Cu(II)/Fe(III) and ascorbate) may further strengthen the anticancer therapeutic potential of redox active cationic *ortho* isomeric Mn(III) *N*-alkyl-alkoxyalkylpyridylporphyrins.

## Figures and Tables

**Table 1:** The values of initial rates of HA degradation, derived from the time-dependent changes in dynamic viscosity (*η*) of the HA solution subjected to MnTE-2-PyP^5+^ or MnTM-4-PyP^5+^ at different concentrations and 100 µM ascorbate in the in the presence of 1 µM Cu(II). Initial rates −(d*η*/d*t*)*_t_*_=0_: were listed where Mn porphyrins were added at the start of HA degradation; rates −(d*η*/d*t*)*_t_*_=60_: Mn porphyrins were added after 60 min of HA degradation.

Mn Complex	0 µM	5 µM	20 µM	100 µM
	**Initial rates −(d*η*/d*t*)*_t_*_=0_/mPa** **·s** **·min^−1^**			
MnTE-2-PyP^5+^	0.0209	0.0244	0.0434	0.0954
MnTM-4-PyP^5+^	0.0311	0.0163	0.0085	0.0074
	**Rates at *t* = 60 min, −(d*η*/d*t*)*_t_*_=60_/mPa** **·s** **·min^−1^**			
MnTE-2-PyP^5+^	0.0354	0.0354	0.0597	0.1282
MnTM-4-PyP^5+^	0.0597	0.0401	0.0190	0.0190

**Table 2:** The values of the rates at 20 min of the process of HA degradation, −(d*η*/d*t*)*_t_*_=20_ are listed and are derived from the time-dependent changes in dynamic viscosity (*η*) of the HA solution subjected to MnTE-2-PyP^5+^ at different concentrations and 100 µM ascorbate in the presence or in the absence of 1 µM Cu(II). Initial rates, −(d*η*/d*t*)*_t_*_=0_: the rate at *t*~0 min.

	Rates at *t* = 20 min −(d*η*/d*t*)*_t_*_=20_/mPa·s·min^−1^			
MnTE-2-PyP^5+^	0 µM	5 µM	20 µM	100 µM
0 µM Cu(II)	0	0.006	0.017	0.115
1 µM Cu(II)	0.020	0.045	0.075	0.215
	**Initial rates at *t* ~ 0 –(d*η*/d*t*)*_t_*_=0_/mPa·s·min^−1^**			
MnTE-2-PyP^5+^	0 µM	5 µM	20 µM	100 µM
0 µM Cu(II)	0	0.006	0.0331	0.0779

## Data Availability

The data used to support the findings of this study are included within the main text.

## References

[1] Batinic-Haberle I., Tovmasyan A., Spasojevic I. (2018). Mn porphyrin-based redox-active drugs: Differential effects as cancer therapeutics and protectors of normal tissue against oxidative injury. Antioxid. Redox Signal..

[2] Batinic-Haberle I., Tome M.E. (2019). Thiol regulation by Mn porphyrins, commonly known as SOD mimics. Redox Biol..

[3] Batinic-Haberle I., Spasojevic I. (2019). 25 years of development of Mn porphyrins—From SOD mimics to thiol signaling to clinical trials. The story of our life in USA. J. Porphyr. Phthalocya..

[4] Batinic-Haberle I., Benov L., Spasojevic I., Hambright P., Crumbliss A.L., Fridovich I. (1999). The relationship between redox potentials, proton dissociation constants of pyrrolic nitrogens, and in vitro and in vivo superoxide dismutase activities of manganese(III) and iron(III) cationic and anionic porphyrins. Inorg. Chem..

[5] Batinic-Haberle I., Benov L., Spasojevic´ I., Fridovich I. (1998). The ortho effect makes manganese(III) meso-tetrakis-(N-methylpyridinium-2-yl)porphyrin a powerful and potentially useful superoxide dismutase mimic. J. Biol. Chem..

[6] Ferrer-Sueta G., Batinic-Haberle I., Spasojevic I., Fridovich I., Radi R. (1999). Catalytic Scavenging of Peroxynitrite by Isomeric Mn(III) N-Methylpyridylporphyrins in the Presence of Reductants. Chem. Res. Toxicol..

[7] Batinic-Haberle I., Reboucas J.S., Spasojevic I. (2010). Superoxide dismutase mimics: Chemistry, pharmacology, and therapeutic potential. Antioxid. Redox Signal..

[8] Weitner T., Kos I., Mandić Z., Batinić-Haberle I., Biruš M. (2013). Acid–base and electrochemical properties of manganese meso(ortho- and meta-*N*-ethylpyridyl)-porphyrins: Voltammetric and chronocoulometric study of protolytic and redox equilibria. Dalton Trans..

[9] Yimcharoen M., Kittikunnathum S., Suknikorn C., Nak-on W., Yeethong P., Anthony T.G., Bunpo P. (2019). Effects of ascorbic acid supplementation on oxidative stress markers in healthy women following a single bout of exercise. J. Int. Soc. Sports Nutr..

[10] Pehlivan F.E. (2017). Vitamin C: An antioxidant agent. Vitamin C.

[11] Valachova K., Topolska D., Mendichi R., Collins M.N., Sasinková V., Soltes L. (2016). Hydrogen peroxide generation by the Weissberger biogenic oxidative system during hyaluronan degradation. Carbohydr. Polym..

[12] Phaniendra A., Jestadi D.B., Periyasamy L. (2015). Free radicals: Properties, sources, targets, and their implication in various diseases. Ind. J. Clin. Biochem..

[13] Valachova K., Hrabarova E., Priesolova E., Nagy M., Banasova M., Juranek I., Soltes L. (2011). Free-radical degradation of high-molecular-weight hyaluronan induced by ascorbate plus cupric ions. Testing of bucillamine and its SA981-metabolite as antioxidants. J. Pharm. Biomed. Anal..

[14] Buffone A., Weaver V.M. (2020). Don’t sugarcoat it: How glycocalyx composition influences cancer progression. J. Cell Biol..

[15] Lahir Y.H. (2016). Understanding the basic role of glycocalyx during cancer. J. Rad. Cancer Res..

[16] Pardoll D.M. (2012). The blockade of immune checkpoints in cancer immunotherapy. Nat. Rev. Cancer.

[17] Mitchell M.J., King M.R. (2014). Physical Biology in Cancer. 3. The role of cell glycocalyx in vascular transport of circulating tumor cells. Am. J. Physiol. Cell Physiol..

[18] Soltes L., Stankovska M., Brezova V., Schiller J., Arnhold J., Kogan G., Gemeiner P. (2006). Hyaluronan degradation by copper(II) chloride and ascorbate: Rotational viscometric, EPR spin-trapping, and MALDI–TOF mass spectrometric investigations. Carbohydr. Res..

[19] Tovmasyan A., Sampaio R.S., Boss M.K., Bueno-Janice J.C., Bader B.H., Thomas M., Reboucas J.S., Orr M., Chandler J.D., Young-Mi Go J.M. (2015). Anticancer therapeutic potential of Mn porphyrin/ascorbate system. Free Radic. Biol. Med..

[20] Valachova K., Rapta P., Batinic-Haberle I., Soltes L., Kulkarni S., Rawat N.K., Haghi A.K. (2021). Manganese porphyrins as pro-oxidants in high-molar-mass hyaluronan oxidative degradation-possible implications in anticancer effects. Green Chemistry and Green Engineering. Processing Technologies, Properties and Applications.

[21] Lokeshwar V.B., Mirza S., Jordan A. (2014). Targeting hyaluronic acid family for cancer chemoprevention and therapy. Adv. Cancer Res..

[22] Batinic-Haberle I., Spasojevic I. (2014). Complex chemistry and biology of redox-active compounds, commonly known as SOD mimics, affect their therapeutic effects. Antioxid. Redox Signal..

[23] Tong Q., Weaver M.R., Kosmacek E.A., O’Connor B.P., Harmacek L., Venkataraman S., Oberley-Deegan R.E. (2016). MnTE-2-PyP reduces prostate cancer growth and metastasis by suppressing p300 activity and p300/HIF-1/CREB binding to the promoter region of the PAI-1 gene. Free Radic. Biol. Med..

[24] Flórido A., Saraiva N., Cerqueira S., Almeida N., Parsons M., Batinic-Haberle I., Miranda J.P., Costa J.G., Carrara G., Castro M. (2019). The manganese(III) porphyrin MnTnHex-2-PyP^5+^ modulates intracellular ROS and breast cancer cell migration: Impact on doxorubicin-treated cell. Redox Biol..

[25] Batinic-Haberle I., Tovmasyan A., Huang Z., Duan W., Du L., Siamakpour-Reihani S., Cao Z., Sheng H., Spasojevic I., Secord A.A. (2021). H_2_O_2_–driven anticancer activity of Mn porphyrins and the underlying molecular pathways. Oxid. Med. Cell. Longev..

[26] Kato Y., Ozawa S., Miyamoto C., Maehata Y., Suzuki A., Maeda T., Baba Y. (2013). Acidic extracellular microenvironment and cancer. Cancer Cell Int..

[27] Dvoranová D., Barbieriková Z., Brezová V. (2014). Radical Intermediates in Photoinduced Reactions on TiO_2_ (An EPR Spin Trapping Study). Molecules.

[28] Spasojevic I., Batinic-Haberle I., Fridovich I. (2000). Nitrosylation of manganese(II) tetrakis(*N*-ethylpyridinium-2-yl)porphyrin. Nitric Oxide.

[29] Bonnesœur S., Morin-Grognet S., Thoumire O., Le Cerf D., Boyer O., Vannier J.-P., Labat B. (2020). Hyaluronan-based hydrogels as versatile tumor-like models: Tunable ECM and stiffness with genipin-crosslinking. J. Biomed. Mat. Res..

[30] Dovedytis M., Liu Z.J., Bartlett S. (2020). Hyaluronic acid and its biomedical applications: A review. Eng. Regen..

[31] Soltes L., Valachova K., Mendichi R., Kogan G., Arnhold J., Gemeiner P. (2007). Solution properties of high-molar-mass hyaluronans: The biopolymer degradation by ascorbate. Carbohydr. Res..

